# The Biopolymer Active Surface for Optical Fibre Sensors

**DOI:** 10.3390/polym16152114

**Published:** 2024-07-25

**Authors:** Karol A. Stasiewicz, Wiktor Bereski, Iwona Jakubowska, Rafał Kowerdziej, Dorota Węgłowska, Anna Spadło

**Affiliations:** Faculty of Advanced Technologies and Chemistry, Military University of Technology, 2 Kaliskiego St., 00-908 Warsaw, Poland; wbereski311099@gmail.com (W.B.); iwona.jakubowska@wat.edu.pl (I.J.); rafal.kowerdziej@wat.edu.pl (R.K.); dorota.weglowska@wat.edu.pl (D.W.); anna.spadlo@wat.edu.pl (A.S.)

**Keywords:** fibre-optic sensor, biopolymer, DNA complex, photonics, optical fibre taper technology

## Abstract

Optical fibre sensors have the potential to be overly sensitive and responsive, making them useful in various applications to detect the presence of pollutants in the environment, toxic gasses, or pesticides in soil. Deoxyribonucleic acid (DNA) as biopolymer active surfaces for fibre sensors can be designed to detect specific molecules or compounds accurately. In the article, we propose to use an optical fibre taper and DNA complex with surfactant-based sensors to offer a promising approach for gas detection, including ammonia solution, 1,4 thioxane, and trimethyl phosphate imitating hazardous agents. The presented results describe the influence of the adsorption of evaporation of measured agents to the DNA complex layer on a light leakage outside the structure of an optical fibre taper. The DNA layer with additional gas molecules becomes a new cladding of the taper structure, with the possibility to change its properties. The process of adsorption causes a change in the layer’s optical properties surrounding a taper-like refractive index and increasing layer diameter, which changes the boundary condition of the structure and interacts with light in a wide spectral range of 600–1200 nm. The research’s novelty is implementing a DNA complex active surface as the biodegradable biopolymer alignment for optical devices like in-line fibre sensors and those enabled for hazardous agent detection for substances appearing in the environment as industrial or even warfare toxic agents.

## 1. Introduction

Fibre sensor technology is still increasingly important for medical, environmental, and industrial monitoring applications or security manufacturing. The classification of fibre sensors into various categories depends on the quantity of substance or analyte to be measured. The main challenge in the optical fibre-derived sensor system technique is an evanescent field sensing observed in the case of tapered optical fibres. The fading wave is created at the sample’s interface when light passes through the optical fibre due to total internal reflection. This field fades exponentially with distance from the interface, and tapered optical fibres are often used in various optical transduction processes, such as refractive index change, absorption, or fluorescence. They efficiently capture recognition signals by the transmission process and convert them to electrochemical, electrical, or optical signals. Additional attributes of tapered optical fibres are increasing sensitivity, shorter response time, transducer performance, reproducibility, and low detection limits for individual molecules [1].

Sensor technologies have improved people’s daily lives with their applications in almost all fields. Such devices detect changes in the source, collect the signals, and give a response. Several sources can be used, including light, temperature, chemical compounds, biological materials, motion pressure, etc. A wide range of applications are being exploited using innovative sensor technologies. Currently, the most advanced real-time detection technologies are fibre-optic sensors, plasmon resonance sensors, resistive sensors, and resonant acoustic wave sensors. The detecting components of fibre-optic sensors are obtained using optical fibres made of glass, polymer, or sapphire, depending on the application [2]. Such sensors have advantages such as immunity to electromagnetic interference, light weight, small size, high sensitivity, large bandwidth, and ease of signal light transmission [3]. Plasmon resonance sensors exhibit extraordinary sensitivity based on the effects of surface plasmon resonance (SPR) or localised surface plasmon resonance (LSPR) [4,5,6]. Surface plasmons are collective electron oscillations that occur at the interface between a metal and a dielectric, generating surface plasmon polaritons upon excitation by light. The use of optical fibres for SPR sensing [7], where changes in the detected parameters (e.g., refractive index) are determined by measuring the intensity distribution of the transmitted spectrum of the white light, enables the detection of refractive index changes in the operating range of 1–1.7 RIU with a resolution of up to 5 × 10^−5^ RIU at higher analyte refractive indices with a resonance wavelength resolution of 0.5 nm [8]. Resistive sensors are used in many applications, such as thermal and light detectors, gas presence/concentration evaluation, position measurement, and strain estimation [9]. Such a sensor provides information in the form of the electrical resistivity of a material or the electrical resistance of a device. The resonant acoustic wave sensors are very sensitive to disturbances and surface changes. Therefore, they measure parameters such as temperature, pressure, liquid density, liquid viscosity, electrical conductivity, mass changes, and viscoelasticity of thin films [10,11,12,13] in both gaseous and liquid environments.

Fibre-optic sensors based on a biopolymer active layer are promising alternatives to conventional ones. Sensors using biopolymers for chemical sensing have progressed and shown helpful perspectives and potential applications. The classification of such sensors is based on the mode of physiochemical transduction or the type of biorecognition element. The operation mode of optical sensors is based on the occurrence of a biochemical reaction, which relates to light absorption or emission. Sensors detect the presence of pesticides, potentially toxic elements, toxins, pathogens, and chemical compounds. The main focus is on quantifying long-lasting toxicants [14].

Combining sensors with biopolymers is a natural and sustainable alternative to petroleum-based polymers. Modern sensors are characterised by high sensitivity, selectivity, on-time response, stability, and reversibility and are low-cost and easy to use. Their great advantage is the quick reaction to the presence of the monitored factor, which allows for quick intervention [15].

The wide range of applications of biopolymers results from their properties, such as their capability to collect and accumulate analytes on sensor surfaces and the possibility of producing various forms (film, sponge, and hydrogel). Biopolymers can be divided into three groups based on the nature of repeating monomer units (polysaccharides, polynucleotides, and polypeptides). Polynucleotides like deoxyribonucleic acid (DNA) and ribonucleic acid (RNA) are long-chain molecule biopolymers of nucleotide units, and the biological nature of the polymer degrades naturally. Biopolymer materials are sustainable and renewable and perform excellently with a low carbon footprint. They are made of monomeric units, which are covalently bonded together to form large structures. Biopolymer complexes have been reported to show higher stability to varying temperatures, pH, and ionic strength. Due to their renewability, abundance, biodegradability, and other unique properties, such as high adsorption capabilities and ease of functionalisation, they are a potential sustainable replacement for petroleum materials [16].

DNA in natural form does not dissolve in the organic solvents typically used to produce photonic polymer devices. Moreover, the DNA thin layers do not achieve sufficient optical quality for applications in photonic waveguides. Therefore, additional processing steps are required to modify the properties and structure of the DNA to make it functional for photonic applications. The key is chemical structure modifications to form a complex of DNA with a cationic surfactant. Additionally, surfactants can influence the self-assembly of DNA into higher-order structures by interacting with the DNA molecules. This reaction has been shown to form a water-insoluble DNA-based biopolymer complex of high optical and thermal quality (thermal stability approx. 200 °C) and suitability for photonic applications [17,18,19].

The optical properties of complex DNA films mainly depend on parameters such as the type of surfactant and the length of the DNA. Using cationic surfactants in DNA reactions requires optimising surfactant concentrations and controlling reaction conditions (temperature, pH, and concentration of the substrates). DNA condensation or aggregation may occur depending on the amount of added surfactant. Another side effect is the neutralisation of the DNA charge, which may affect its migration and solubility. In addition, destabilisation of the DNA structure may occur by breaking hydrogen bonds between nitrogenous bases, which leads to DNA denaturation. Some cationic surfactants may also act as inhibitors of enzymatic reactions, including DNA polymerisation, which reduces reaction efficiency [20,21].

The DNA thin-layer complex for photonic applications is usually generated from quaternary ammonium-based surfactants such as cetyltrimethylammonium chloride (CTMA). It exhibits high transmission in the near-infrared and visible regions of the spectrum. The refractive indices of these thin films vary broadly with wavelength but remain constant in the range of 1000–1250 nm, rendering them as low-loss optical materials [22]. The novelty of the proposed research is the performance of DNA structure modification by other cationic surfactants to stabilise the DNA complex and verify its useful properties.

The present work explores the potential of using the proposed DNA complex as an active layer for optical fibre taper, emphasising specific interactions with various detected substances and the ability to transform optical transmission.

## 2. Materials and Methods

Creating new sensing devices based on fibre-optic technology is often associated with enhancing reception or modifying the boundary conditions for the propagating light beam. In this article, the base element is an optical fibre taper that allows the leakage wave to interact with the external environment. The additional materials will be described in the following section to enhance or allow the interaction of the leakage beam, the use of which is necessary.

### 2.1. Synthesis of Biopolymer

**Figure 1:**
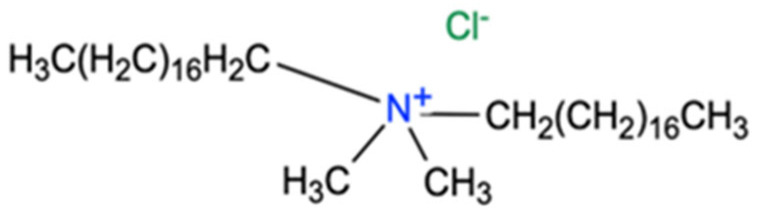
The cationic surfactant structure of dimethyldioctadecylammonium chloride (DODA).

A cationic surfactant, namely quaternary ammonium chloride with two long alkyl chains (Figure 1), was selected to optimise the required structure of the DNA complex.

The surfactant choice considers the already-known connections between the length of the surfactant’s alkyl chain and the properties of the obtained biopolymer. The length of the alkyl chain of the surfactant hydrophobic group influences the formation of the DNA complex. Increasing the length of the surfactant’s alkyl chain changes the properties of the complex created, resulting in reduced solubility in aqueous solutions. However, the solubility of the complex in organic solvents increases, which is an important application element in thin-film production technology. Additionally, it increases the packing of surfactant molecules at the interfacial surface (if the area occupied by the hydrophilic group allows it) and increases the thermal stability of the complex [20].

The DNA–surfactant complex was synthesised based on the procedure described previously [23]. An additional advantage of the process and production of a functional biopolymer is the environmental aspect related to the possibility of waste disposal, low cost, and a simple DNA modification reaction scheme.

The material for synthesis was obtained in the form of DNA sodium salt from Across Organics. The source of DNA is factory salmon waste. DNA sodium salt specification: MW = 50.000–100.000 Daltons (Da) purity above 96%. DNA sodium salt and surfactant DODA (MW = 586.5 g/mol, purity > 97%; Sigma-Aldrich, Co., St. Louis, MO, USA) were dissolved in deionised water. A white precipitate of the resulting DNA complex with the DODA surfactant was obtained. The obtained DNA-DODA complex was dissolved in ethanol (50 mL) to obtain a 4% solution. The structure of the complex was confirmed by the IR spectroscopy method. Then, an appropriate technological line was prepared to produce fibre-optic sensors with the active layer of the obtained complex.

Creating a DNA complex involves replacing sodium ions in DNA salts with surfactant ions. As a result, a new connection is created based on electrostatic interactions with the DNA phosphate group. As a result of strong hydrophobic interactions between the surfactant chains, the long alkyl chains align in appropriate positions to the biopolymer layer. The alkyl chains of the surfactant are oriented perpendicular to the biopolymer layer. The achiral DNA helices are oriented parallel to the plane due to electrostatic attraction [24].

### 2.2. Experimental Materials

This paragraph describes the materials used to create the device and enable different compound detections.

As a basis for creating new sensors, standard telecommunication optical fibres of SMF-28 from Corning^®^ (Corning, NY, USA) type were selected. This type of fibre is related to its parameters and well-developed processing technology. This fibre-optic cable is inexpensive, and the parameters are standardised, allowing for direct connection with most available measuring equipment. The optical fibres have a core diameter of about 8 µm, a cladding of about 125 µm, and a mode field diameter of about 10 µm. Wavelengths above 1280 nm are characterised by single-mode operation with less than 0.2 dB/km efficiency. Below this wavelength, propagation becomes multimode with a simultaneous increase in the diameter of the modulus field, which directly participates in detection. The principles of propagation in optical fibres are widely described in the literature [25]. It should be noted that the core and cladding material is glass-doped with rare earth particles (core one to increase the refractive index RI).

**Figure 2:**
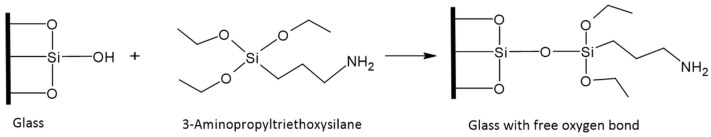
Scheme of reaction of preparation of glass structure with APTES.

One work noted that the same DNA-DODA complex, even though it has good adhesion properties [17] for the flat glass surfaces in the case of the cylindrical surfaces of the taper, requires additional assistance—activation of the surface of the taper waist. The second part of, therefore, APTES ((3-aminopropyl) triethoxysilane)) was used as a coupling agent. The main and most important parameters describing APTES should be mentioned: boiling temperature 217 °C/760 mmHg, density 0.946 g/mL at 25 °C, and purity 99%. Figure 2 presents a scheme for connecting APTES to the glass structure [26,27].

After applying APTES (about 150 s), the isopropanol was applied to the surface to interrupt its impact. After activation of the taper surface, the last part of the sensor manufacturing was related to applying the DNA-DODA complex layer in the deep coating process. The present work focused on detecting three types of liquids administered to the vaporisation process and their adsorption by the DNA-DODA complex.

Our investigation focused on three liquids that can influence people’s health. The table below provides a brief description of the compounds analysed. In this work, we subjected three substances to study: 1,4-thioxane (THX), trimethyl phosphate (TMP), and ammonium hydroxide (NH_4_OH). One of the deciding factors was the significant difference in the vapour pressure of these compounds. The other was their toxicity/harmfulness. THX and TMP are imitators of the chemical warfare agents organosulfur (e.g., sulphur mustard) and organophosphorus (e.g., sarin), respectively, which have similar physical and chemical properties but are safer and permitted for use. Ammonium hydroxide, on the other hand, is used in household cleaners, photography and fertilisers, textiles, rubber, and pharmaceuticals and is also used as a refrigerant and can result in serious damage to the lungs (inhalation) as well as the skin and eyes (contact with the liquid). Characterisation of the main parameters of the mentioned compounds is presented in Table 1.

Creating new miniaturised fibre-optic sensors requires selecting the appropriate materials mentioned above, including optical fibres as an element propagating the beam, which will change parameters under the influence of external factors and materials that allow a change in the boundary conditions affecting the beam. In the next paragraph, the set-up for measurement, taper element manufacturing, and special arrangement allow for proper layering of the materials mentioned above.

## 3. Measurements

### 3.1. Optical Fibre Taper Element Manufacturing

The process of manufacturing a sensing device involves a few parts. The first are those related to manufacturing a tapered fibre structure as the base of all-optical devices.

**Figure 3:**
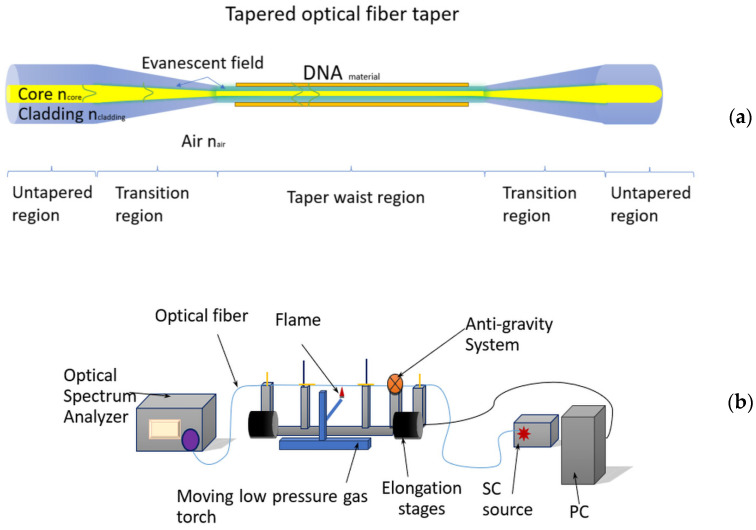
(**a**) Scheme of TOF with the main region and (**b**) scheme of the used FOTET.

As it is well known, a standard fibre light propagates inside the core structure, and only a small part propagates in a cladding part as an evanescence wave [28,29,30,31]. There is no possibility to interact with it directly. For direct access to the propagating beam, the boundary conditions in the fibre must be changed. One of the methods developed at the MUT Institute of Technical Physics is a rewiring process widely described in the literature [31,32] in the Fiber Optic Taper Element Technology (FOTET) set-up. This process consists of heating the fibre in a non-pressurised mixture of propane–butane oxygen and then, after the softening temperature of the fibre has been reached, pulling it out until it gains a suitable diameter, for which the diameter of the modulus field fills the entire taper structure. Such an obtained structure is called the tapered optical fibre (TOF). For our investigation, a series of TOFs were characterised by low losses below α = 0.2 dB in the whole investigated range. This corresponds to the adiabatic taper profile, ensuring the least possible losses and a smooth transition between the untapered region and the taper optical waist region. In our investigations, TOFs were elongated to the length L = 20.20 ± 0.05 mm, which corresponds to a taper waist diameter of φ = 14.50 ± 0.50 μm. Such a taper possesses a double-clad structure [32]. In this structure, the diameter of the core is almost negligible; the original standard cladding becomes the first part of the cladding, providing low losses; and the air surrounding the structure becomes a new second cladding, which can be easily replaced with other materials that are sensitive and adsorb external factors by changing chemical (composition) and optical (absorption of light radiation, refractive index, and dimension of layer) properties. The most interesting from the sensing point of view to mention materials like graphene [32], liquid crystals [33], polymers [28], or the DNA-DODA complex, which was used in this study. Figure 3a presents the main regions of the manufactured taper and Figure 3b the scheme used for the set-up.

As shown in Figure 3, an adiabatic taper with low losses is divided into three main regions: taper waist, transition region, and untapered region. The transition region converts optical light from the untapered region to the taper waist and conversely. All measurements relate to the taper waist region, where the evanescence wave can interact with external materials (creating double clad), which is sensitive to different factors. The presented scheme of the FOTET system (Figure 3b) has been widely described in previous articles [16,20]. Here, it should be mentioned that its main advantage is the anti-gravity system, which allows for breaking the taper into its final part, working on all kinds of fibre and for manufacturing different shapes of tapers. The TOF parameters mentioned above were due to experimental experiments and the selection of the most favourable propagation parameters, including loss and mechanical parameters, i.e., susceptibility to rupture in further processing concerning the preparation of the sensor. This arrangement allows for the security and assembly of the created taper for additional use, including the coating with an active DNA layer that is proposed in this paper. The second part of the manufacturing device is connected to applying active sensing material.

### 3.2. System of Deposition Thin Layers and Measurement Arrangements

**Figure 4:**
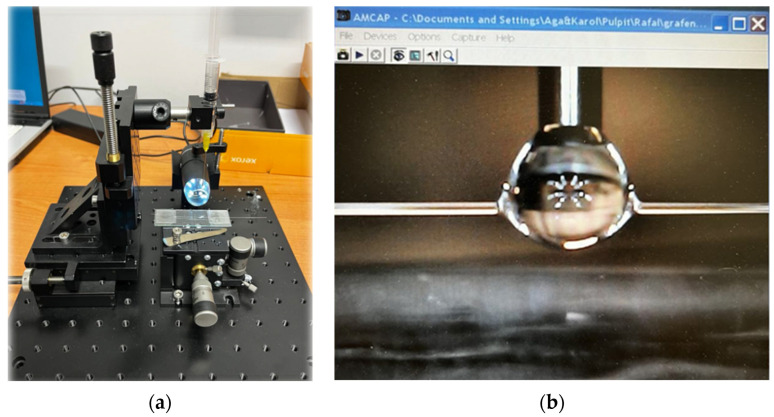
(**a**) The set-up for deep coating of tapered optical fibre; (**b**) the application process of a drop of DNA-DODA complex.

The preparation of new sensors based on the combination of the DNA-DODA complex as a thin layer with fibre-optic technology requires the proper preparation of fibre-optic surfaces to enable the correct adhesion of the DNA-DODA complex layer. Figure 4 presents a picture of the arrangement for the deep coating procedure for APTES, isopropanol, and DNA complex.

Using a charge-coupled device (CCD) camera, it is possible to observe the coating process in live view and enable application on the chosen location of TOF. The deep coating was obtained by a syringe with a 0.8 mm needle diameter and a flat tip. The diameter of the applied drop was around 1.5 mm. Such dimensions allow for application only for the chosen part of the taper waist and interaction between the DNA-DODA layer and compounds.

**Figure 5:**
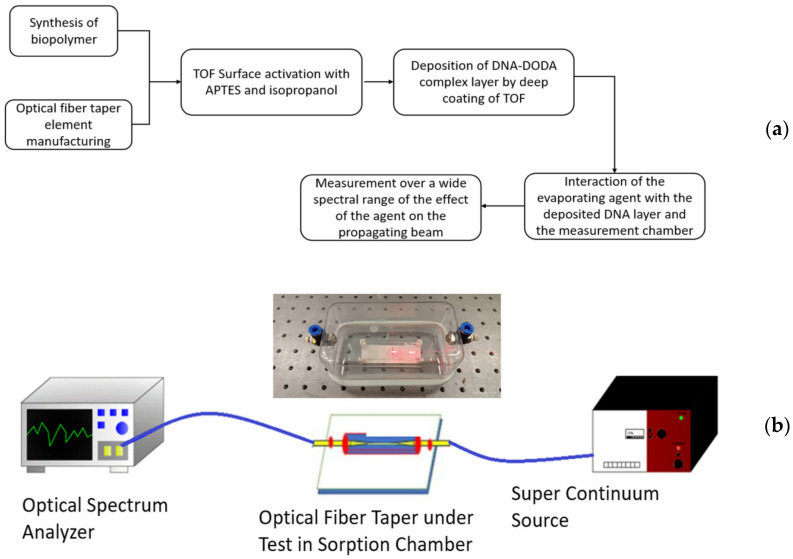
(**a**) A flowchart for the manufacturing sensors process is presented in the top picture, (**b**) and a measurement scheme of the prepared set-up with a picture of a sorption chamber is presented at the bottom.

Such prepared sensing devices with the active sensing layer of the DNA-DODA complex were subjected to the influence of different compounds. The measuring chamber enabled the use of gasses and vapours of liquids measured in the described article. Figure 5a presents a flowchart for manufacturing sensors. Exposure to the mentioned compounds was provided in a measurement set-up, as shown in the scheme in Figure 5b.

As a light source in the transmission measurement system, a broadband light source, SuperK Extreme EXR-15 (NKT Photonics, Birkerod, Denmark; in the 400–2400 nm range), was used. From the wide range after preliminary use, the VIS range was chosen in which the highest changes can be observed. The choice of the visible band was dictated by the much greater interaction between the applied layer and the given wavelength range. For the 600–1200 nm range, the leakage field is much larger than for the lengths for which the fibre from which the TOFs were made, which is single-mode. The special splitter connected to the NKT PhotonicsSuperK SPLIT laser range VIS/NIR, enabled for the selected range, was used. As a detector, the optical spectral analyser AQ6373 (YOKOGAWA, Tokyo, Japan) operates in the 350–1200 nm range. For all measurement compounds, an equal volume of 100 µL was used. The chamber developed and built for the study presented in the picture above has a capacity of 0.8 dm^3^. In it was placed a tapered optical fibre with a layer of DNA-DODA complex applied, and a glass vessel containing 100 µL of the compound to be analysed was placed directly below the taper. The measurements carried out were correlated with the properties of the studied evaporating liquids and their physical and chemical properties, including vapour pressure volatility and adhesion to the DNA-DODA complex layer. Measurements were performed at constant room temperature and atmospheric pressure. Following the preliminary studies conducted and determination of the properties of the agents tested, measurements were set from 1 min to 120 min, in which the sensor’s performance and its response to the substances tested were examined. The sensor was mounted on a special pedestal and faced directly toward the evaporating substance. The OSA system additionally averaged the measurements collected.

## 4. Results and Discussion

### 4.1. IR Spectra

**Figure 6:**
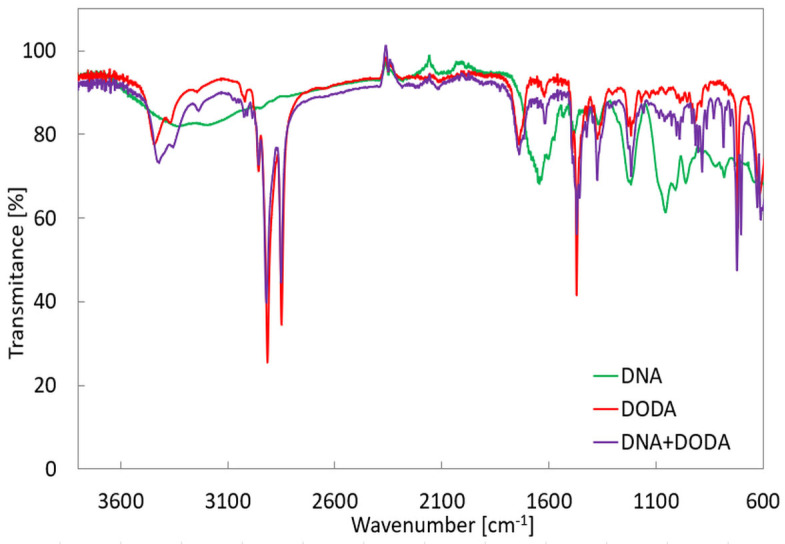
The infrared spectra of investigated compounds. Green: DNA, red: DODA, and purple: complex DNA with surfactant DODA.

Infrared spectra of the DNA, DODA, and complex DNA_DODA measurements with reflective mode from crystals using attenuated total reflection (ATR) technique with diamond crystal were carried out, and the Nicolet iS10 Thermo Scientific spectrometer (Thermo Fisher Scientific, Waltham, MA, USA) was used for collection. The spectra were recorded using a diamond crystal within a wavenumber range of 400–4000 cm^−1^ with a resolution of 2 cm^−1^ from 24 scans. Before each sample measurement, the background measurement was recorded. The comparison of the IR spectra of DNA, DODA, and the DNA-DODA complex are presented in Figure 6. The spectra presented in Figure 6 are average spectra from five random points on the surface of each sample.

Several strong vibrational bands at 1060, 1200, 1400, and 1650 cm^−1^ were observed, which are specific for pure DNA’s infrared (IR) spectra [34]. The pure DNA spectrum also signs a broad absorption band around 3000–3400 cm^−1^, assigned to molecular vibrations: N–H-stretching modes, C=N vibrations, and OH symmetric and antisymmetric stretching modes. The bands in the 1400–1800 cm^−1^ range show nucleobase vibrations, and the band at 1400 cm^−1^ is assigned to C=C-stretching vibration, which is attributed to purine imidazole ring vibration. Compared to the DNA spectrum, the following absorption bands appeared in the DODA surfactant and complex DNA with DODA surfactant: 2920 and 2850 cm^−1^. These bands correspond to symmetric and asymmetric stretching C–H vibrations of the −CH2 and −CH3 groups. The peak observed at 1472 cm^−1^ in the DODA surfactant and the complex spectrum corresponds to symmetric stretching C–H vibrations of −CH2 and −CH3 groups. These peaks confirm that the surfactants are bound to the DNA in the complex sample.

### 4.2. Atomic Force Microscope Topography

**Figure 7:**
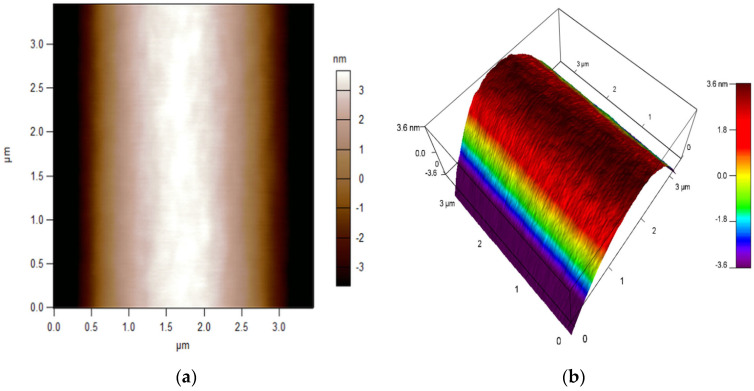
AFM characterisation of optical fibre’s surface topography: (**a**) 2D image; (**b**) 3D image.

**Figure 8:**
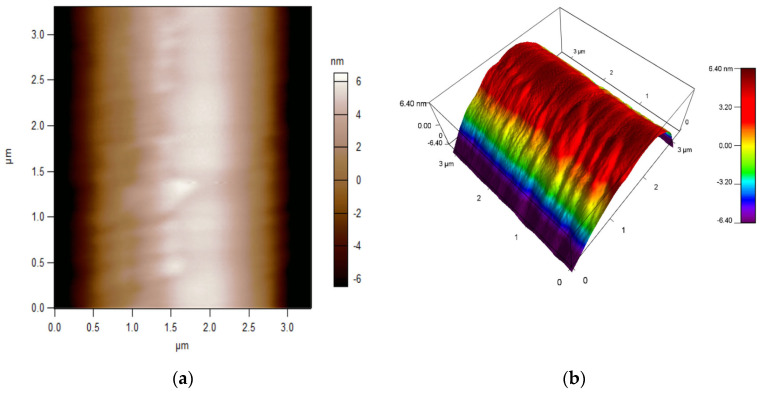
AFM characterisation of optical fibre with DNA layer’s surface topography: (**a**) 2D image; (**b**) 3D image.

The initial part of the measurement focused on validating the properties related to the deposition of the active layer. Atomic force microscope (AFM) microscopy was utilised for the research, which aimed to characterise the surface topography and evaluate the homogeneity of the DNA layer applied. Firstly, the surface of the optical fibre was characterised and, subsequently, the optical fibre with the DNA layer. The findings are illustrated in Figure 7 and Figure 8, respectively. The topography was examined using the fast-scanning atomic force microscope Cypher (Oxford Asylum Research, Santa Barbara, CA, USA), with conventional micro cantilever OMCL-AC160TS-R3 (Olympus, Tokyo, Japan, Oxford Instruments, Abingdon, UK). The OMCL-AC series has a tetrahedral tip on the exact end of the cantilever. The nominal probe spring constant was equal to 26.1 N/m, and the tip radius was 7 nm. Maps showing the surface topography of pure optical fibre and after DNA application were acquired by adopting a contact mode, and then, they were extracted graphically using the IGOR Pro ver. 6.32A, software provided by the AFM supplier. AFM measurements proved that the roughness of a pure optical fibre expressed in the root mean square (RMS) is RMS = 3.206 nm and that applying the DNA-DODA complex to its surface increases it to RMS = 4.844 nm.

The first part of the AFM measurements was to collect references and characteristics for a pure optical fibre. The results are presented in Figure 7.

The second part of the investigation was related to measuring the dimension of the deposited DNA layer on a standard fibre.

Based on the above measurements, it was concluded that the proposed method enables DNA-DODA layering and allows for further research.

### 4.3. Propagation of the Light Beam in the Fabricated Structures

The measurements were carried out for a series of fabricated fibre-optic sensors. The first step was to perform reference measurements in a chamber without the analysed agent to eliminate differences that fibre-optic constrictions may have (differences in connection, insertion losses, etc.) as well as differences related to the dip-coating procedure of each layer.

Measurements were performed for each sample at intervals of 0, 1, 2, 3, 4, 5, 30, 60, and 120 min from exposure to the analysed agent. The choice of such timepoints was related to previous data measurements and the literature references [35,36,37] on the reaction time of THX, TMP, and aqueous ammonia solution with sensitive layers and the evaporation rate. A series of measurements was carried out for each agent to obtain reproducible results, and the selected results for the liquid evaporation data are presented below.

**Figure 9:**
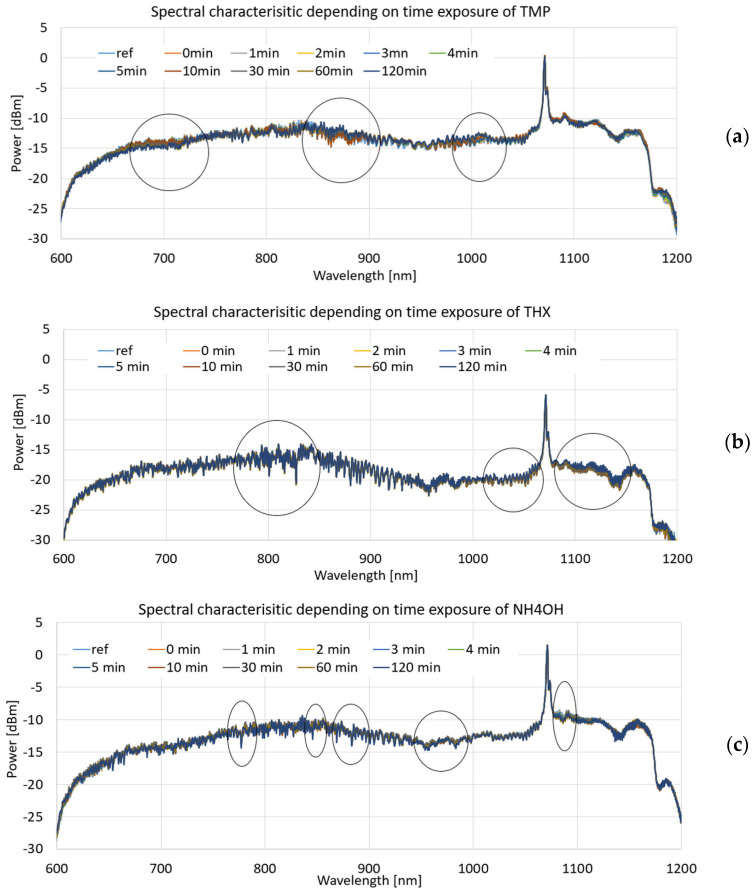
Spectral characteristics depending on time exposure for 0 to 120 min to (**a**) TMP compound, (**b**) THX, and (**c**) as a result for NH_4_OH. Results contain characteristics for the optical range 600–1200 nm, with a mark showing the highest change of power.

Figure 9 presents the full spectral characteristics in a range of 600–1200 nm for the mentioned time exposure to an agent, with a mark showing the highest observable change of characteristics, for which we calculated the fastest change of power depending on time of exposure to the agent. Figure 9a presents the spectral characteristics for the TMP compound, Figure 9b for THX, and Figure 9c for NH_4_OH.

Power deviations for the results obtained were within ±0.5 dBm for spectral measurements over the entire spectral range.

For the next part, we present the spectral differences characteristics of TOFs coated with a DNA-DODA layer exposed to the mentioned agents of TMP, THX, and NH_4_OH at 1 min, 5 min, 30 min, and 120 min for different wave ranges of 600–800 nm, 800–1000 nm, and 1000–1200 nm to show how this layer reacts with vapouring compounds.

Power deviations connected with differential calculations were obtained within 0.005 dBm for selected spectral ranges.

**Figure 10:**
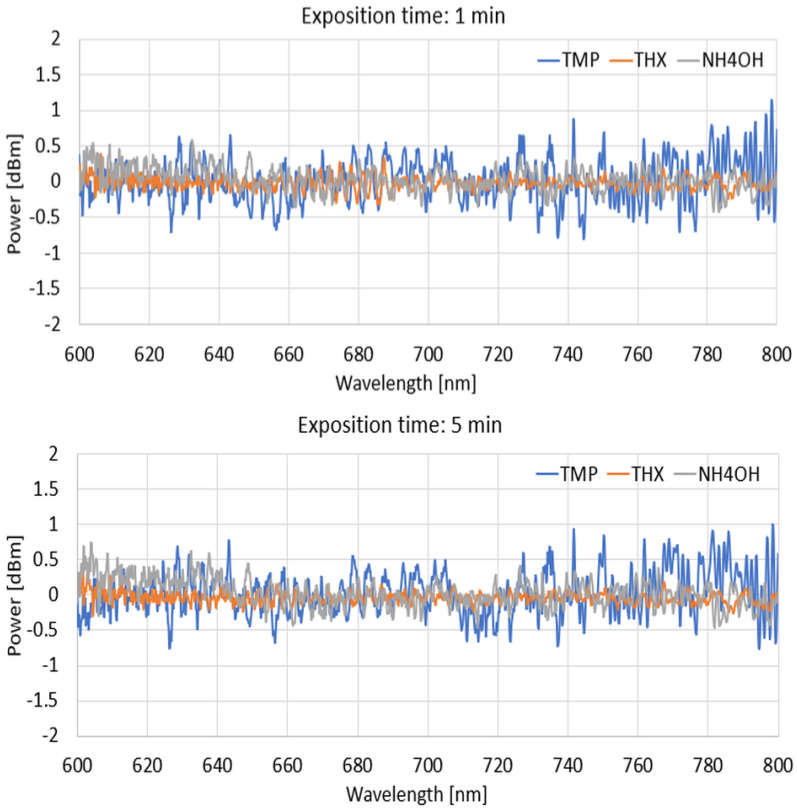

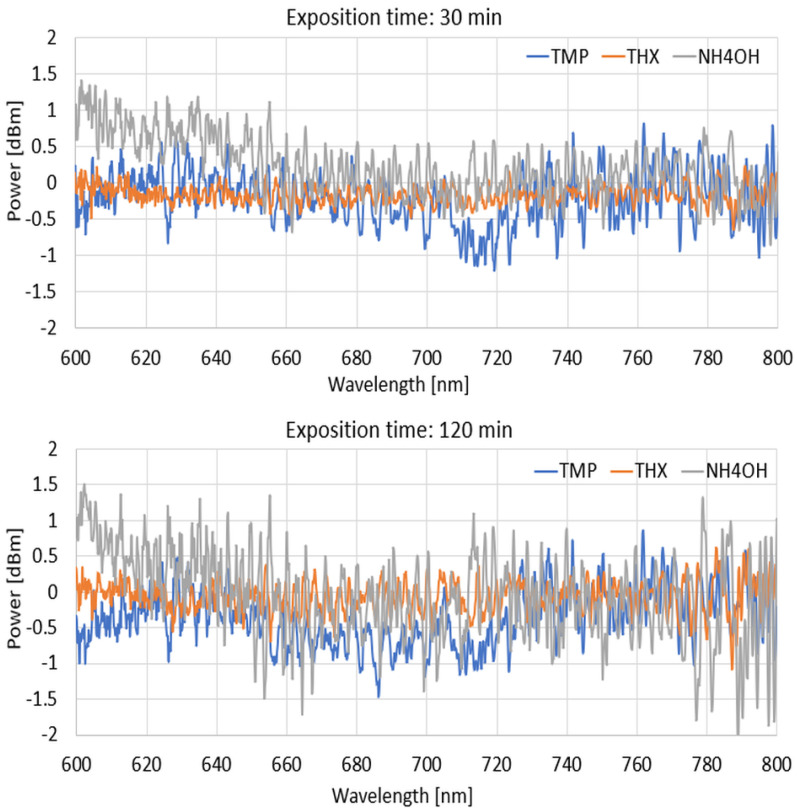
Spectral differences in characteristics range from 600 to 800 nm for 1, 5, 30, and 120 min.

**Figure 11:**
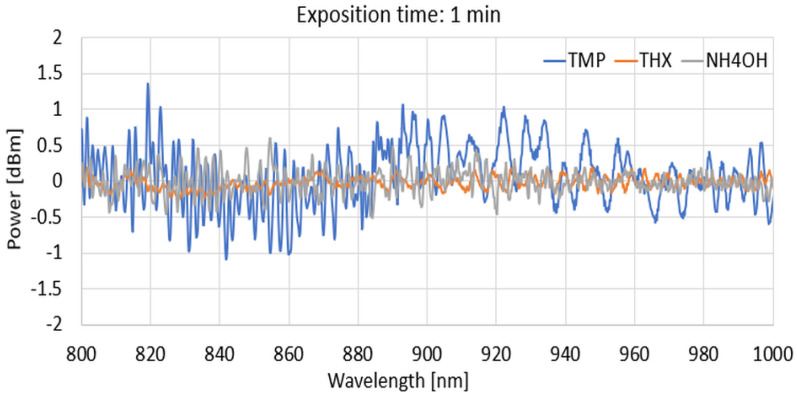

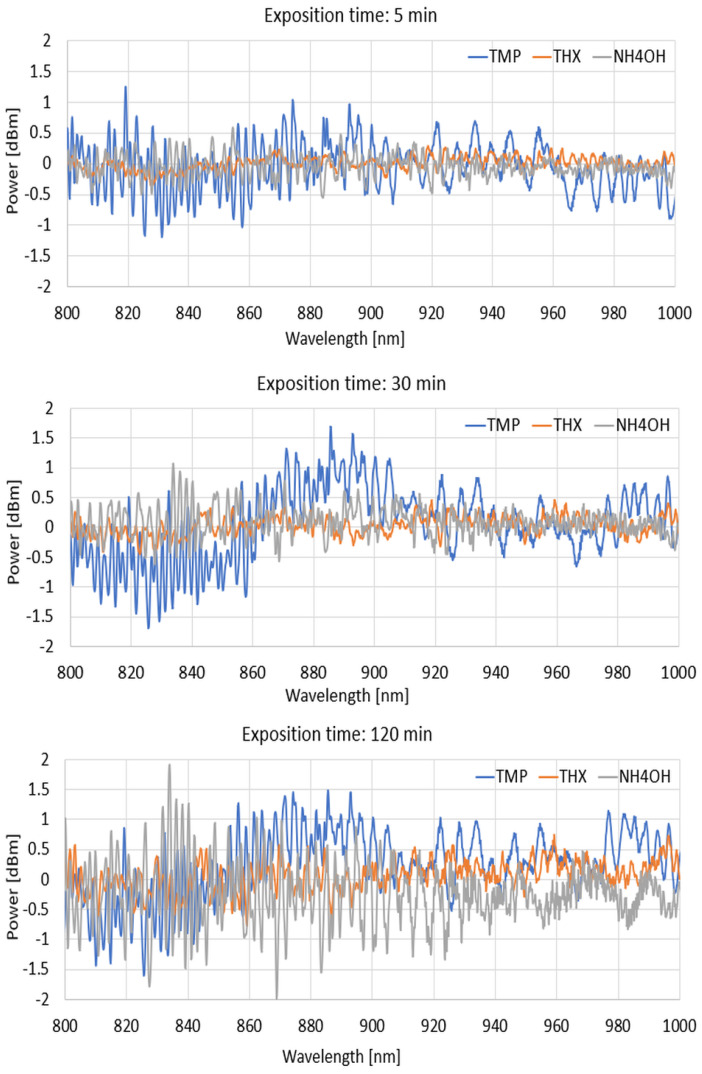
Spectral differences in characteristics range from 800 to 1000 nm for 1, 5, 30, and 120 min.

**Figure 12:**
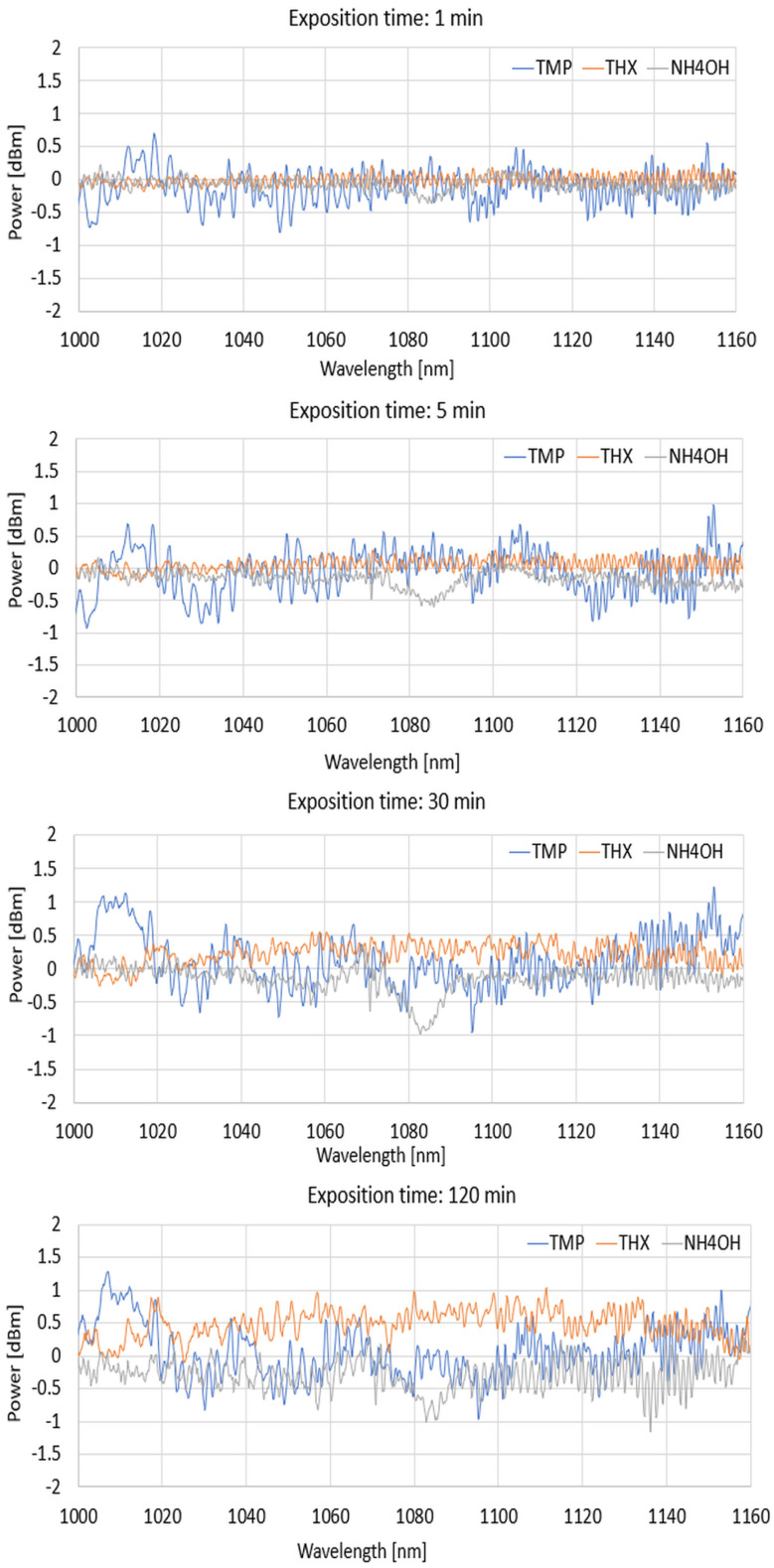
Spectral differences in characteristics range from 1000 to 1200 nm for 1, 5, 30, and 120 min.

From the above graphs for narrowed wavelength ranges, it can be deduced that each of the compared factors produces different spectral characteristics from which it is possible to identify which material is being analysed at a given time or which factor is acting on the sensitive layer, changing its optical properties. Evaporation of the mentioned agents causes changes in the thickness of the new layers, which is the effect of the adsorption of agents on the DNA-DODA. A newly formed layer changes the propagation conditions for all wavelengths in each range. Analysing the studied range allows the selection of the most suitable wavelengths for which the dynamics of change are greatest. It directs additional studies to determine the speed/rate of adsorption of the measured TMP, THX, or NH_4_OH factors relative to the DNA-DODA material in a given spectral range. Narrowing the measured ranges to 200 nm, as shown in Figure 10, Figure 11 and Figure 12, one can see more clearly the differences in the individual agents’ spectral characteristics and adsorption rates to the DNA-DODA layer. Both the occurrence of single wavelengths, for which significant enhancement/weakening with time of exposure to the agents is seen, or certain spectral ranges (TMP: 1000–1020 nm; NH_4_OH: 1070–1090 nm; TMP: 1000–1200 nm), for which the change in power is significant compared to the layer without exposure to the agents in question, are also evident.

From the resulting characteristics, the change in signal level per minute was calculated for the 1 min, 5 min, 30 min, and 120 min times listed above. According to the marked areas in Figure 9, for each factor, four characteristic peaks or the middle peak in the mentioned range were selected from the area for which calculations were made of changes in power concerning one minute. The results for agent THX are shown in Table 2, for TMP in Table 3, and NH_4_OH in Table 4. Results are presented as an absolute value.

From the measurements and calculation included in Table 2, Table 3 and Table 4, it can be seen that for NH_4_OH, the largest changes began after about 30 min for exposure to the mentioned factors, especially in an NIR range; for TMP, the largest changes were in the range of 5–30 min, and then, stabilisation of the characteristics was observed. For THX, higher changes were observed for the NIR range, which significantly accelerated after 30 min, and the maintenance rate grew to 120 min. As can be noticed, the factor of transmitted light is different for different materials. In all kinds of gasses/evaporating liquids, it is strictly possible to diversify the spectrum depending on the material reacting with the DNA-DODA layer.

## 5. Conclusions

The DNA-DODA complex was obtained with desirable optical properties and increased chemical and thermal stability. A thin layer of the DNA-DODA complex was created on the optical fibre by the coating technology process, which meets the characteristics of an active optical medium for detecting the presence of gases in the environment. The experiments confirmed theoretical predictions regarding using the DNA-DODA complex as an active layer for optical fibre sensors. DNA structure surface modification techniques were used, and interactions with various target molecules were examined, which allowed the sensitivity and specificity of the sensors to be assessed. Applying a DNA-DODA layer to the fibre-optic taper makes it possible to measure various factors, including the evaporation of different liquids, including aqueous ammonia solution, 1,4-thioxane, or trimethyl phosphate. After analysing the results and the answers we obtained due to the differences in the spectral characteristics of these compounds and their physicochemical properties and affinity for the sorbent, it is possible to separate the information about the detected compound. Also important are the times when the maximum sorption of each tested material for a given wavelength are reached. In the future, we will attempt to measure the response of our solution to mixtures of test compounds. The measurement conducted over a wide spectral range showed that for each of the tested compounds, there is a response in different spectral ranges. The layers for each agent were tested in different ways, absorbing the materials and forming a new thin layer with different refractive indexes, directly influencing light propagation. An additional advantage of that biopolymer structure is biodegradability, and the synthesis is ecological and low-cost from an economic point of view. DNA sensors can be designed to bind to target molecules specifically. The use of fluorescence and other molecular recognition techniques can significantly enhance the sensitivity of these sensors, allowing for the detection of target molecules. Our work contributes to the field in the following ways: (1) exploring the integration of DNA with other robust materials (e.g., graphene, carbon nanotubes) to enhance mechanical strength and durability while maintaining biocompatibility and functionality; (2) developing integrated systems that combine DNA sensors with other technologies to create multifunctional sensing platforms for complex applications; and (3) expanding the range of detectable targets by exploring new functionalisation techniques and molecular recognition elements, broadening the scope of DNA sensors in various fields.

These results may contribute to developing a new generation of fibre-optic sensors with increased sensitivity and stability, which is crucial in many technological and industrial fields.

## Figures and Tables

**Table 1:** Short characterisation of the tested compounds.

Properties/Characterisation		Compound	
	ammonia solution	1,4-thioxane	trimethyl phosphate
Structural Formula	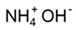	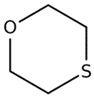	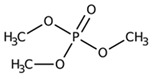
Physical State	liquid	liquid	liquid
Appearance	clear, colourless	clear, colourless	clear, colourless
Odor	strong, irritating, pungent	strong, irritating, pungent	no
Melting Point (°C)/Freezing Point (°C)	−72 °C	30 °C	−46 °C
Boiling Point (°C)	36 °C	147 °C	197 °C
Density (g/cm^3^)	0.965	1.114	1.197
Vapor Pressure (mmHg)	115	5.7	0.415
Refractive Index	1.355	1.509	1.379
Hazards Identification	corrosive; causes severe skin, eye, and digestive tract burns; harmful if swallowed; mist or vapor extremely irritating to eyes and respiratory tract	flammable liquid and vapour; causes skin irritation; causes serious eye irritation; may cause respiratory irritation	harmful if swallowed; causes skin irritation; causes serious eye irritation; may cause genetic defects; suspected of causing cancer

**Table 2:** Calculated signal change for the chosen wavelength for the THX agent.

THX Compound				
Wavelength (nm)	1 min (dBm)	5 min/per min (dBm)	30 min/per min (dBm)	120 min/per min (dBm)
800.8	0.09	0.08/0.016	0.122/0.002	0.027/0.0003
827	0.088	0.117/0.023	0.038/0.003	0.01/0.0001
1019.6	0.057	0.097/0.019	0.275/0.011	0.347/0.004
1102.6	0.031	0.035/0.007	0.184/0.007	0.423/0.005

**Table 3:** Calculation of signal change for chosen wavelength for TMP agent.

TMP Compound				
Wavelength (nm)	1 min (dBm)	5 min/per min (dBm)	30 min/per min (dBm)	120 min/per min (dBm)
719.2	0.088	0.205/0.041	0.667/0.027	0.266/0.003
881	0.063	0.327/0.065	0.655/0.026	0.051/0.0006
983.6	0.089	0.307/0.061	0.869/0.035	0.569/0.006
1010.8	0.04	0.135/0.027	0.73/0.029	0.057/0.0006

**Table 4:** Calculation of signal change for chosen wavelength for NH_4_OH agent.

NH_4_OH Compound				
Wavelength (nm)	1 min (dBm)	5 min/per min (dBm)	30 min/per min (dBm)	120 min/per min (dBm)
601.2	0.052	0.035/0.007	0.876/0.035	0.072/0.008
840.2	0.043	0.023/0.005	0.361/0.014	0.495/0.005
957.4	0.066	0.026/0.005	0.012/0.0005	0.762/0.008
1082.8	0.083	0.319/0.064	0.389/0.015	0.023/0.0003

## Data Availability

The data is available upon request due to the volume of data as well as project limitations.
